# PERCEIVED WORKPLACE AGEISM AND OLDER WORKERS’ LABOR FORCE TRANSITIONS DURING THE GREAT RECESSION

**DOI:** 10.1093/geroni/igad104.1530

**Published:** 2023-12-21

**Authors:** Duygu Basaran Sahin

**Affiliations:** RAND Corporation, Brooklyn, New York, United States

## Abstract

Previous research suggests that workplace ageism increased during the Great Recession (GR). During the GR, despite having lower unemployment rates compared to younger workers, older workers took considerably longer to find jobs once they were unemployed. Yet, no study has investigated the potential role of workplace ageism in older workers’ labor force outcomes. This study examines the role of perceived workplace ageism in older workers’ labor force transitions during the GR using longitudinal data from the Health and Retirement study. I conduct a multi-period analysis and compare how the association of perceived workplace ageism with labor force transitions changed during the GR compared to pre and post-recession periods. Results from multivariate logistic regressions show that only in the GR period, both measures of perceived workplace ageism – pressure to retire before age 65 and preference for young in promotions – were associated with a higher likelihood of employment instability (b= 0.336 p< 0.05 and b= 0.340 p< 0.05), net of demographic, socioeconomic, financial, job-related and health-related factors. Moreover, older workers perceiving workplace were less likely to retire and thereby more likely to be unemployed (b= -0.667 p< 0.05). In contrast, perceived workplace ageism had no association with reducing or increasing the hours worked per week. In sum, this study provided the first individual-level evidence on the impact of perceived workplace ageism on older adults’ labor force transitions during the GR. Findings can be used to motivate additional studies on older workers’ experience during the Covid19 crisis.

